# Propitious temporal changes in clinical outcomes after transcatheter compared to surgical aortic valve replacement; a meta-analysis of over 65,000 patients

**DOI:** 10.1186/s13019-021-01689-3

**Published:** 2021-10-20

**Authors:** Ankur Panchal, Andreas Kyvernitakis, Geetha Rayarao, Mark Doyle, Robert W. W. Biederman

**Affiliations:** 1 Department of Internal Medicine, University of Pittsburgh Medical Center, Pittsburgh, PA USA; 2 Department of Cardiovascular disease, Allegheny General Hospital, Pittsburgh, PA USA

**Keywords:** TAVR, SAVR, Aortic stenosis, Stroke, Atrial fibrillation, Mortality

## Abstract

**Background:**

The treatment of symptomatic severe aortic stenosis (AS) has rapidly evolved over the past decade, in both transcatheter (TAVR) and surgical aortic valve replacement (SAVR), resulting in reported improved clinical outcomes. Operator experience and technical improvements have improved outcomes especially for patients undergoing TAVR. We sought to determine and compare 1-year outcomes using a contemporary meta-analysis.

**Method:**

We searched the Medline (MESH), Cochrane and Google scholar databases using keywords “AS”, “atrial fibrillation” (AFib) and “stroke”. We performed a meta-analysis to compare TAVR with SAVR populations for post-procedural stroke, all-cause and cardiovascular mortality at 1-year.

**Results:**

A total of 23 studies met criteria for analysis with total population of 66,857 patients, of which 61,913 had TAVR and 4944 had SAVR. Temporal trends demonstrated overall improvement in outcome for both, TAVR and SAVR groups through the decade. Outcomes, in terms of stroke (3.1% vs. 5%), all-cause (12.4% vs. 10.3%) and cardiovascular mortality (7.2% vs. 6.2%) were similar at 1-year, in TAVR versus SAVR, respectively.

**Conclusion:**

Despite overall gradual improvement in both TAVR and SAVR outcomes over the decade, there is a statistical overlap in confidence intervals for all-cause, cardiovascular mortality and postprocedural stroke at 1-year. While 23 individual studies demonstrate considerable advantages of each technique in certain cohorts, integrating over 65,000 pts with our stratified surgical analysis suggests that TAVR is comparable to SAVR for low and intermediate risk population while superior to SAVR *only* in the highest-risk population for short and intermediate term outcomes. This has substantial socio-economic implications as we contemplate expanding our TAVR indications to low/intermediate risk populations.

## Introduction

Aortic stenosis (AS) is a progressive debilitating valvular heart disease with rapid development of clinical heart failure and high risk of mortality once patients become symptomatic. Aortic valve replacement is the standard of care for severe symptomatic AS patients and is associated with significant improvement in symptoms, quality of life and survival [1]. Transcatheter aortic valve replacement (TAVR) was introduced in 2002 and, although initially considered as a salvage procedure for patients who are prohibitive surgical risk for surgical aortic valve replacement (SAVR), emerging literature continues to reveal constant progressive technical advancements with improved valve systems, matched with improved implantation hardware and techniques making it an attractive alternative, not only for high-risk but also for selected intermediate and low risk patients [2–4]. Given the significant accumulation of data from trials and cohort studies, we conducted a meta-analysis to determine any clinical differences in adverse clinical outcomes and identify temporal changes between patients undergoing TAVR and SAVR over nearly two decades.

## Materials and methods

### Study design

**Fig. 1:**
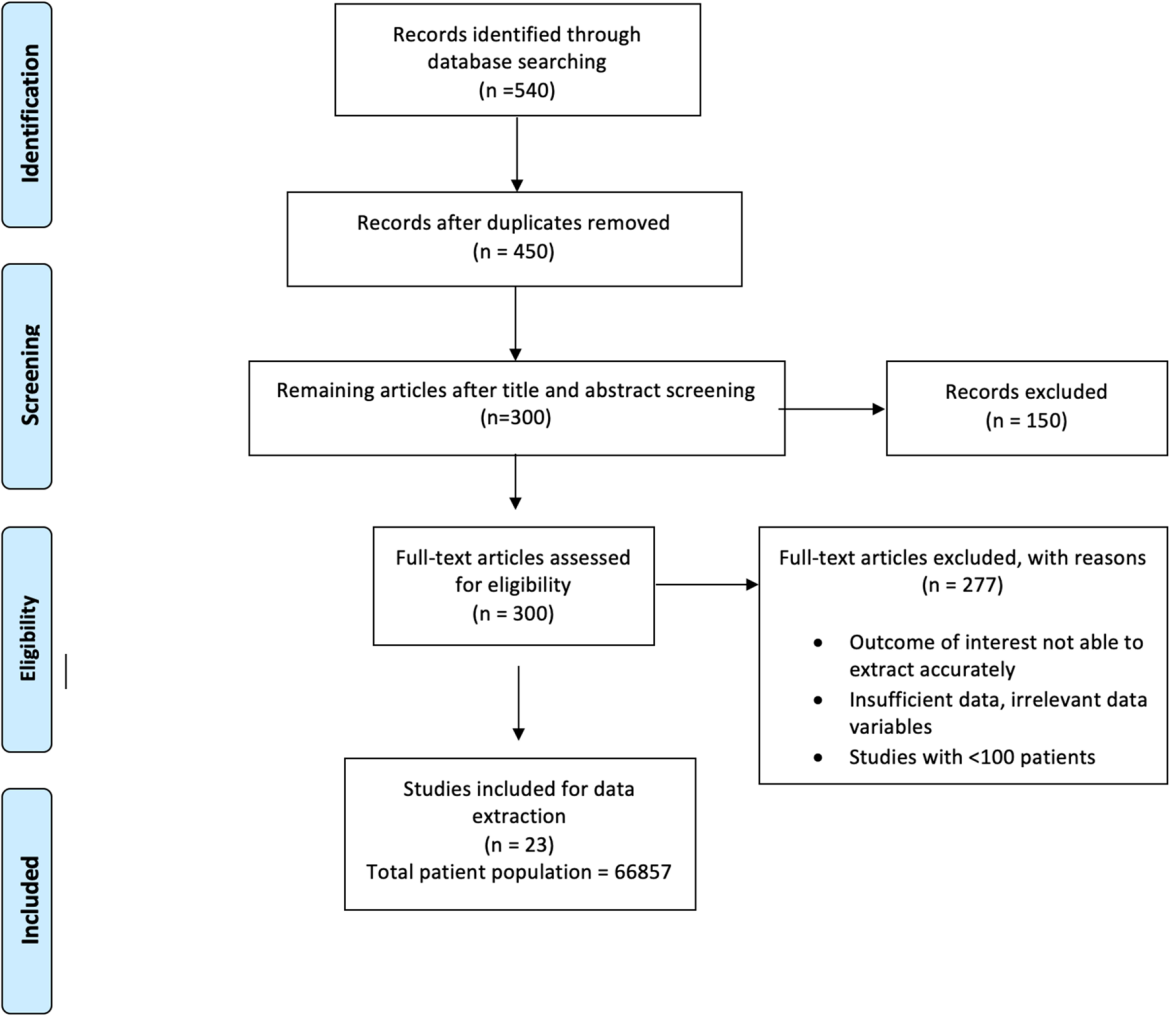
PRISMA diagram for study selection

The study was designed according to the PRISMA (Preferred Reported Items for Systematic Reviews and Meta-analyses) guidelines (Fig. 1) [5]. We carried out an extensive literature search through Medline (MESH), Cochrane and Google scholar databases using the keywords “AS”, “atrial fibrillation” (AFib) and “stroke”, and reviewed TAVR and SAVR studies from January 2005 to November 2018. Two independent investigators assessed the eligibility of the studies for inclusion and divergence was resolved by 3rd reviewer. The risk of bias was evaluated by all authors based on Cochrane collaboration methods for Randomized clinical trials (RCT) and Risk Of Bias In Non-randomized Studies of Interventions (ROBIN-I) for observational studies.

### Inclusion criteria

The included studies were restricted to RCTs, prospective and retrospective studies. Case reports and small case series were not included in our analysis. Overlap of included patients was strictly avoided and duplicate studies were excluded. We included both the Society of Thoracic Surgeons (STS) and the Logistic European System for Cardiac Operative Risk Evaluation (EuroSCORE) system for risk stratification of both groups (TAVR and SAVR) into low, intermediate or high risk for mortality. The adverse clinical outcomes of our study (primary endpoints) included (1) Post-procedural stroke (2) All-cause mortality and (3) Cardiovascular mortality at 1-year. Studies not reporting data on AFib were excluded considering stroke was one of our primary endpoints.

### Statistical analysis

The relative risk (RR) was calculated for grouped results of each outcome. Statistical significance of the differences between TAVR and SAVR groups was performed using meta-analysis (Mantel–Haenszel) with a random-effect model. Heterogeneity of the studies was assessed using Cochran's Q test to calculate I2 and Z test was used to assess the significance of the effect size. The differences between the results in the TAVR and SAVR groups were considered significant if *p* < 0.05. The statistical analyses were performed using the R program meta [6, 7] and SPSS for Windows v 18, IBM Inc. In order to represent the heterogeneity of the studies, we constructed forest plots of the risk ratio. A visual assessment of publication bias was assessed by inspection of funnel plots. Weighting for publication ‘n’ was performed but in the R program meta, additional strengthening of statistical observations was accomplished through accounting for homogeneity, variability and bias.

TAVR and SAVR patient populations were organized as three 4-year groups to analyze the temporal trend and organized by surgical risk to assess variation with risk level. The rate over time and between groups was assessed using Chi-square, with *p* < 0.05 considered statistically significant.

## Results

Out of 540 studies, 23 studies met our eligibility criteria with the majority excluded due to specific reasons (Fig. 1). Eight studies were RCTs, and the remaining were prospective and retrospective studies (Table 1). Overall, we included 66,857 patients from January 2005 to November 2018, among which 61,913 underwent TAVR and 4944 SAVR.

**Table 1:** Included studies

Study name	Type of the study	Total no. of patients
PARTNER (2011) [26]	RCT	699
PARTNER 2A [27]	RCT	2032
PARTNER 3 [28]	RCT	950
NOTION [29]	RCT	280
SURTAVI [30]	RCT	1660
CoreValve Pivotal [2]	RCT	747
Evolut 2019 [31]	RCT	1468
Wakesman et al. [32]	RCT	919
Castrodeza et al. [33]	Observational	362
FRANCE-2 [34]	Observational	3875
SOURCE-XT [35]	Observational	1925
Yankelson et al. [36]	Observational	380
Sannino et al. [37]	Observational	708
Nombela et al. [38]	Observational	1061
Tay et al. [39]	Observational	253
Stortecky et al. [40]	Observational	389
Muneretto et al. [41]	Observational	110
Barbash et al. [42]	Observational	371
Abramowitz et al. [25]	Observational	47,643
Maan et al. [43]	Observational	137
Yoon et al. [44]	Observational	347
Abdelgawad et al. [45]	Observational	143
Zweiker et al. [46]	Observational	398

**Fig. 2:**
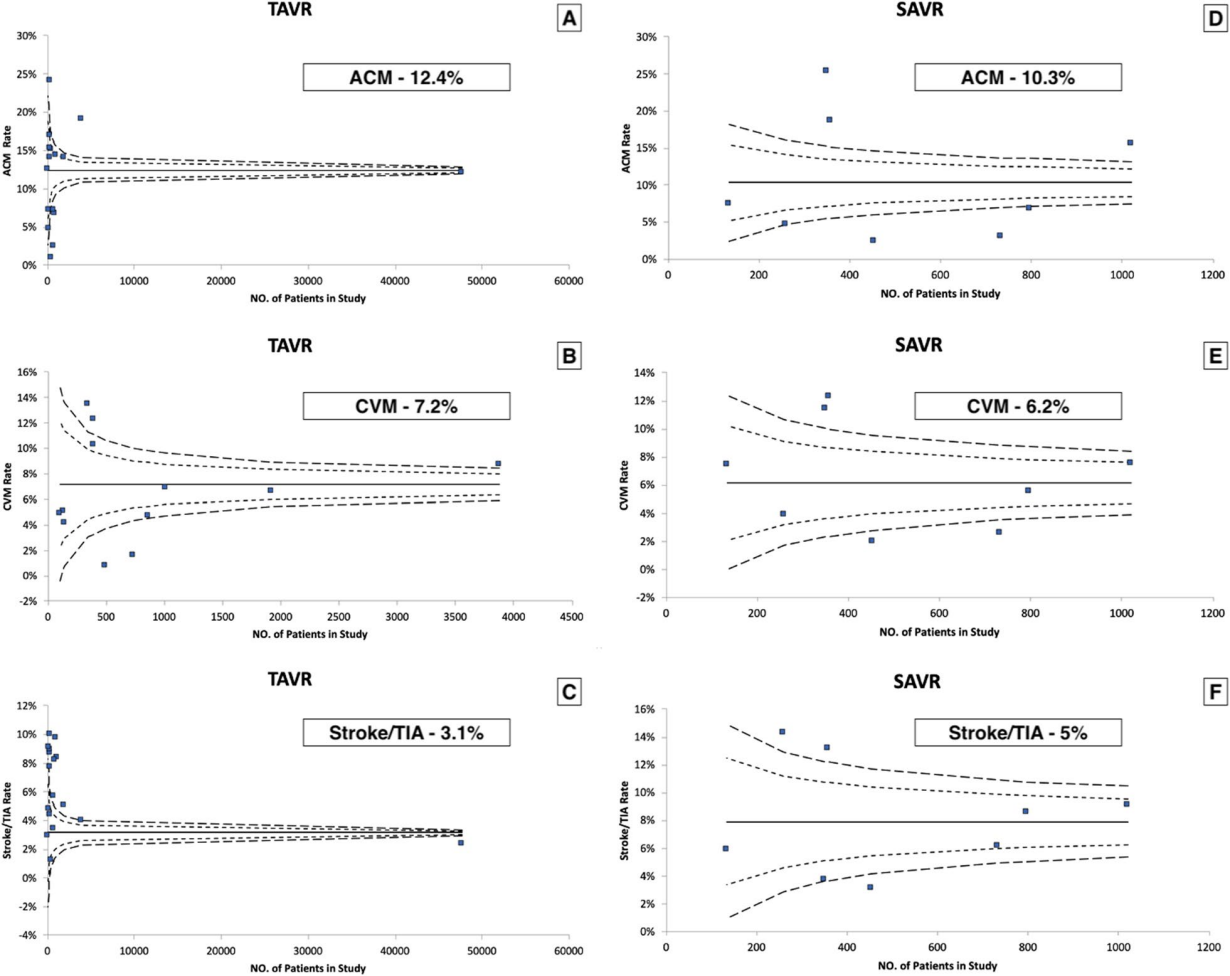
Funnel plot diagram for all-cause mortality at 1-year, cardiovascular mortality at 1 year and stroke/TIA at 1 year (**A**–**F**)

**Fig. 3:**
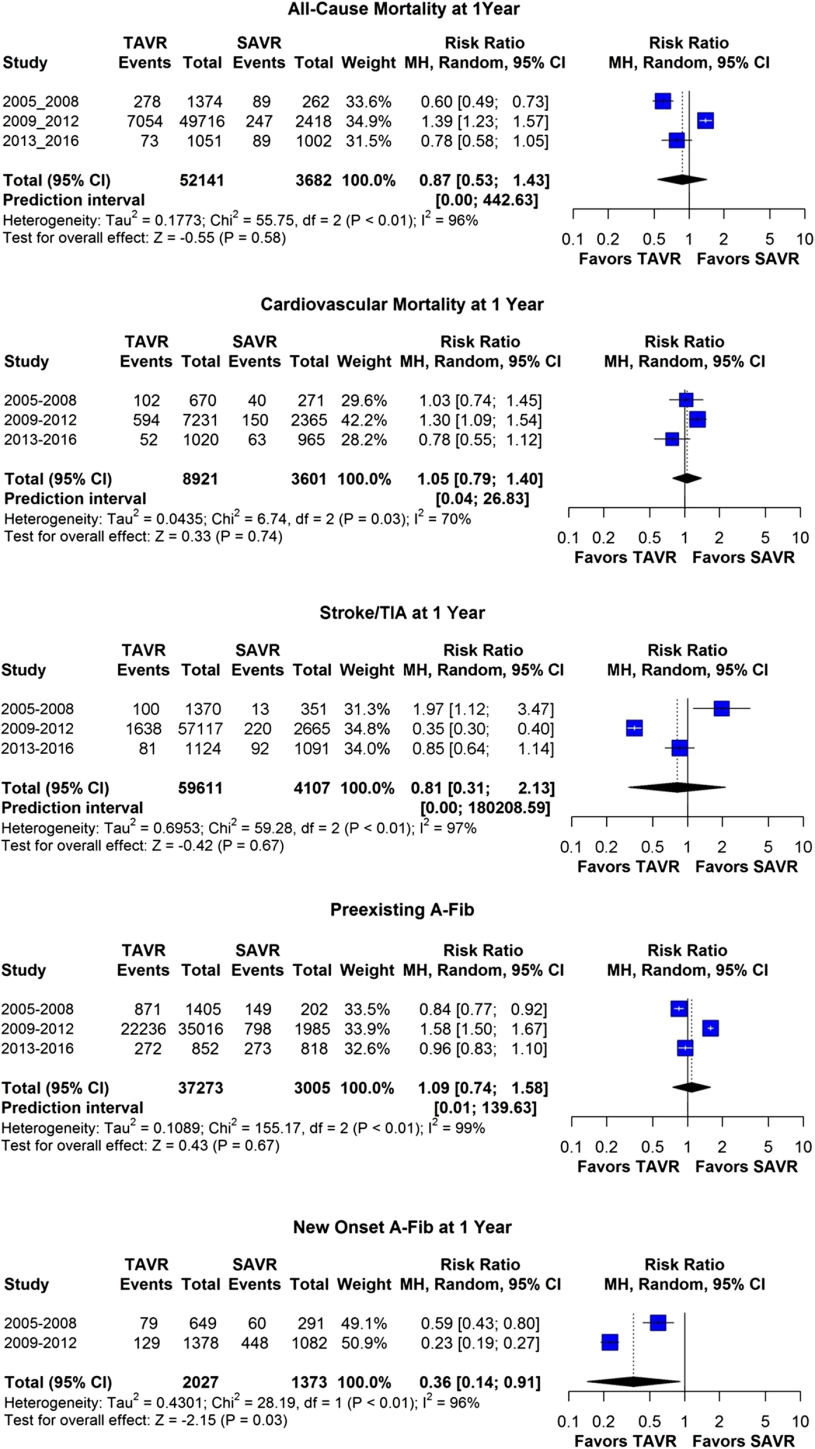
Forest Plot meta-analysis for all-cause and cardiovascular mortality at 1 year, stroke/TIA at 1 year, preexisting AFib at 1 year and new onset AFib at 1 year, stratified by the year group tertile

In our forest plot meta-analysis, overall, post-procedural stroke at 1-year was 3.1% in TAVR versus 5% in SAVR patients (RR, 0.81; 95% CI, 0.31–2.13; *p* = 67). All-cause mortality at 1-year was 12.4% in the TAVR versus 10.3% in SAVR group (RR, 087; 95% CI, 0.53–1.43; *p* = 0.58), whereas CV mortality at 1-year was 7.2% in the TAVR versus 6.2% in the SAVR group (RR, 1.05; 95% CI, 0.79–1.4; *p* = 74) (Figs. 2, 3).

**Fig. 4:**
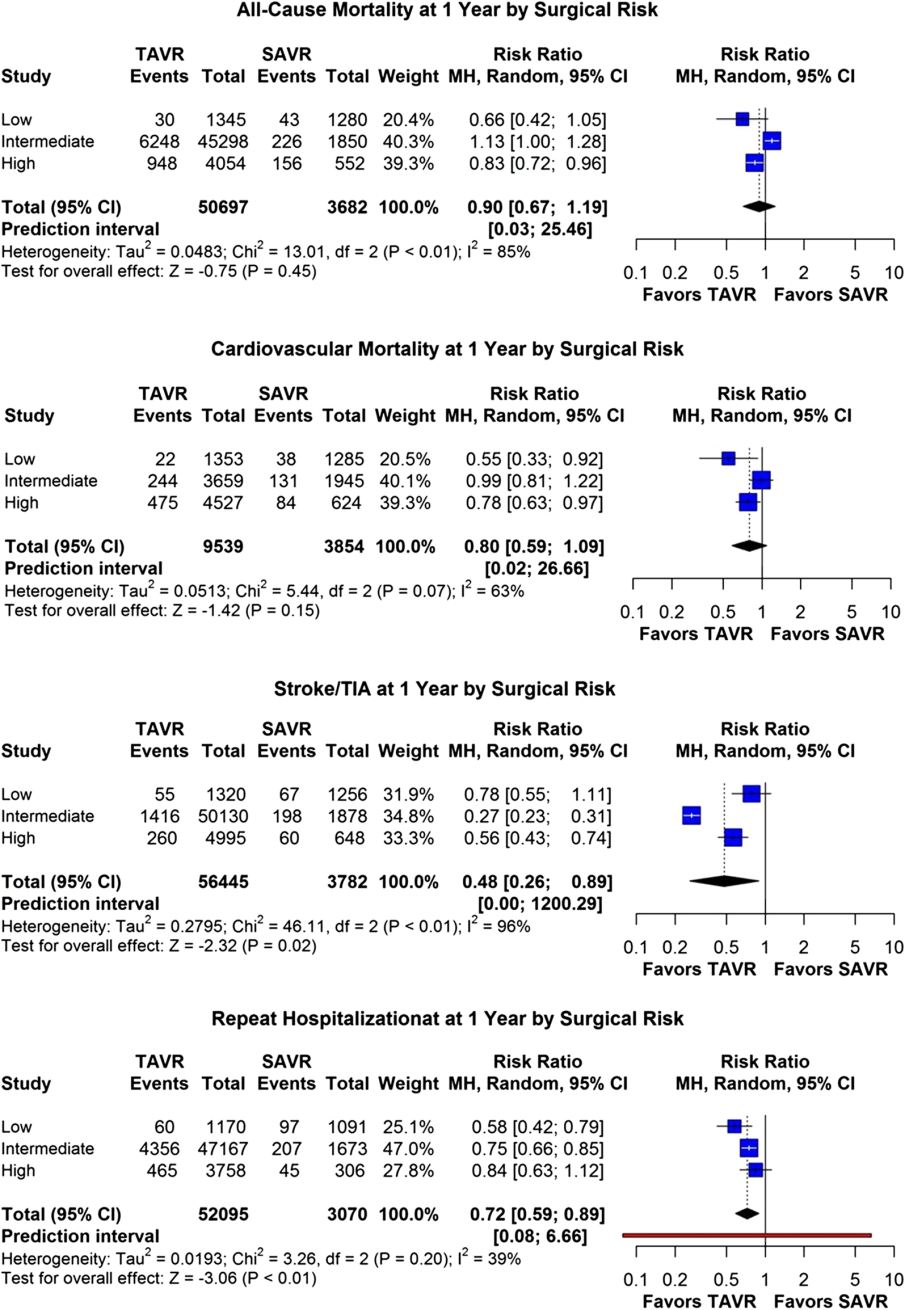
Forest Plot meta-analysis for all-cause and cardiovascular mortality at 1 year, stroke/TIA at 1 year and repeat hospitalization at 1 year, stratified by the surgical risk

**Fig. 5:**
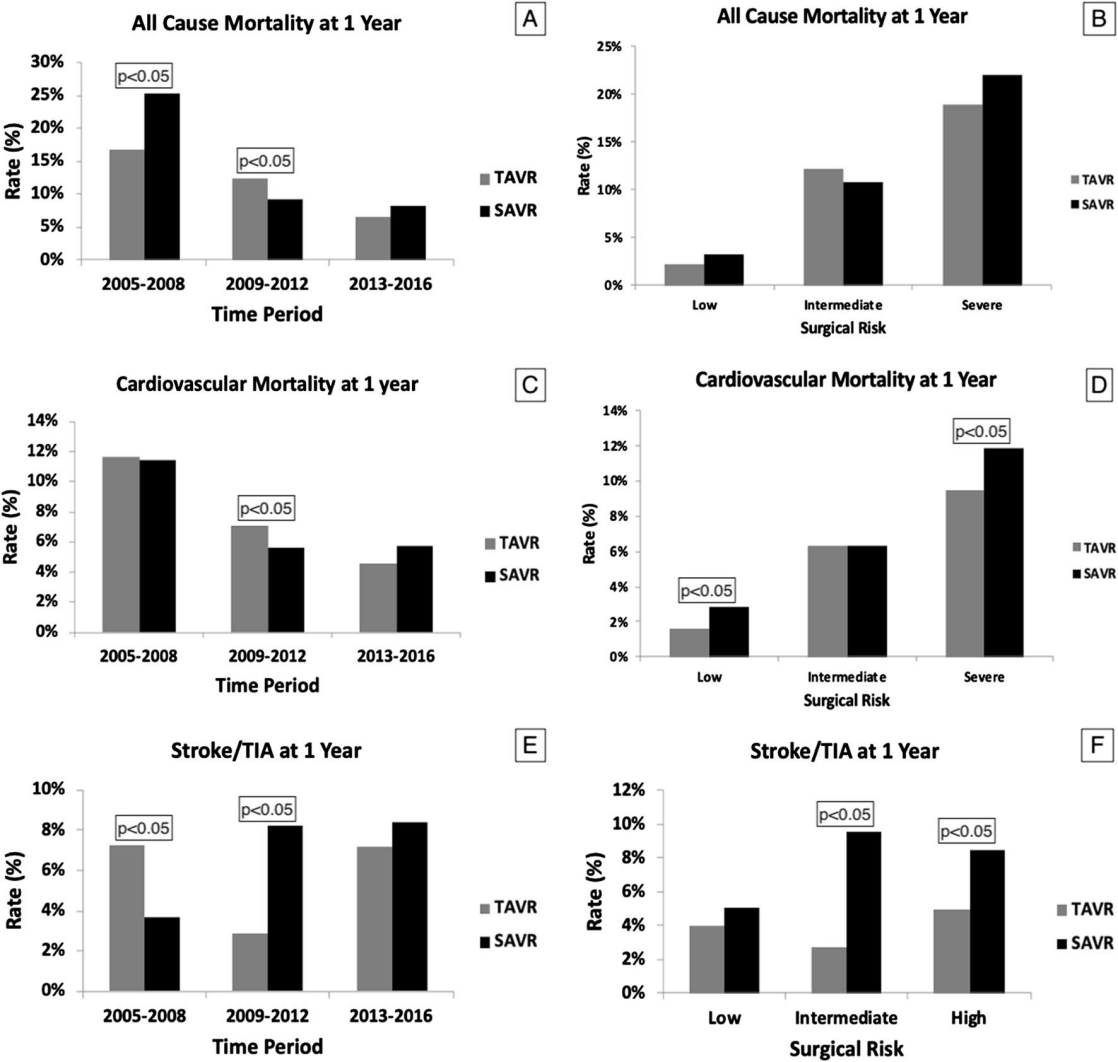
**A**–**F** Temporal changes over the decade for all-cause mortality, cardiovascular mortality and stroke/TIA at 1 year. Increased incidence of these outcomes with increased surgical risk. The changes overtime and the changes between surgical risks are statistically significant (*p* < 0.05)

To study temporal changes between the two groups, we divided our total patient population in three 4-year subcategories. There was a significant temporal decline in all-cause and cardiovascular mortality at 1-year in both groups (TAVR all-cause mortality trend—17 to 12 to 6%; SAVR all-cause mortality trend—25 to 9 to 8%; TAVR cardiovascular mortality trend—11.6 to 7 to 4.6%; SAVR cardiovascular mortality trend—11 to 5.6 to 5.7%; *p* < 0.05 for changes over time for all) (Fig. 5A, C). On the other hand, even though the changes over time were significant (*p* < 0.05) for post-procedural stroke at 1 year for both groups, there was no significant temporal change for TAVR but had progressively higher reported post-procedural stroke for SAVR group (TAVR trend—7 to 3 to 7%; SAVR Stroke/TIA trend 3.7 to 8.2 to 8.7%; *p* < 0.05 only for SAVR) (Fig. 5E).

**Fig. 6:**
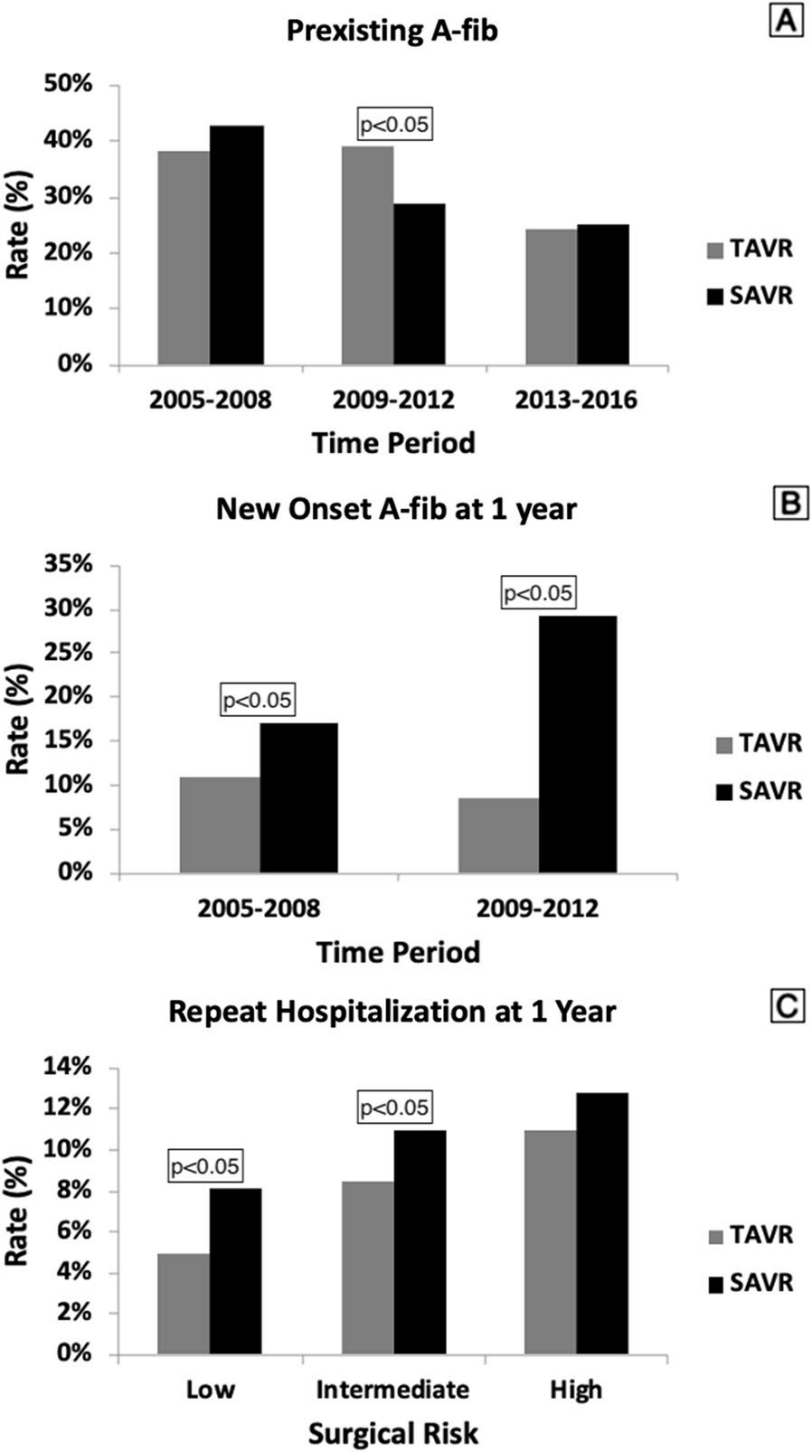
**A** Temporal decline in preexisting AFib over the decade. The changes overtime is statistically significant (*p* < 0.05). **B** Higher risk of new onset AFib with SAVR compared to SAVR and temporal increase in new onset AFib in early decade. The changes overtime is statistically significant for SAVR but not TAVR (*p* < 0.05). **C** Increased rehospitalization with increased surgical risk. The changes between the surgical risks are statistically significant (*p* < 0.05) for figure **C**

Overall, preexisting AFib was more common in TAVR compared to SAVR patients (38% vs. 25%, *p* < 0.0001). Although there was no temporal decline noted in post-procedural stroke at 1-year in either group, there was a decline over time noted in the rates of preexisting AFib in both populations (38 to 24% for SAVR; 42 to 25% for TAVR; *p* < 0.05 for changes over time) (Figs. 5E, 6A). New onset AFib post valve replacement, was progressively higher in the SAVR group compared to the TAVR group (2005–2008 group to 2009–2012 group; 10 to 8% for TAVR, *p* = NS; 17 to 29% for SAVR, *p* < 0.05) and did not change significantly over time in TAVR but did for SAVR (Fig. 6B). In relation to this, we found that the incidence of stroke/TIA at 1-year was higher in SAVR population in 2009–2012 group compared to 2005–2008 group (8.2 to 3.7%; *p* < 0.05) (Fig. 5E). There were no studies reporting data on new onset AFib for our last 4 years of study (2013–2016 group).

When stratified according to the pre-procedural surgical risk, there was a progressive increase in stroke, all-cause and cardiovascular mortality at 1-year with increasing surgical risk in both populations (Figs. 4, 5B, D, F). For *low surgical risk*, there was no statistically significant difference in all-cause mortality and stroke at 1-year between the two groups though cardiovascular mortality at 1-year was significantly lower in TAVR compared to SAVR (1.6% vs. 2.8%, *p* < 0.05) (Figs. 4, 5B, D, F). For *intermediate surgical risk*, there was no statistically significant difference in all-cause and cardiovascular mortality at 1-year, however stroke was significantly lower in TAVR compared to SAVR patients (2.7% vs. 9.5%, *p* < 0.05) (Figs. 4, 5B, D, F). For *high surgical risk*, there was no statistically significant difference in all-cause mortality at 1-year though cardiovascular mortality and stroke at 1-year were significantly lower in TAVR patients (9.5% vs. 11.8%; *p* < 0.05 and 4.9% vs. 8.4%; *p* < 0.05, respectively) (Figs. 4, 5B, D, F). Repeat hospitalizations at 1-year were progressively higher with increasing surgical risk in both groups. Rehospitalization after valve replacement was significantly higher for low (4.8% vs. 8.1%; *p* < 0.05) and intermediate surgical risk (8.4% vs. 11%; *p* < 0.05) patients undergoing SAVR compared to TAVR but there was no difference between groups in high surgical risk patients (Figs. 4, 6C).

## Discussion

Since the advent of TAVR in the early 2000s, there has been a tremendous amount of research and evolution in the field of aortic valve replacement. This led to considerable improvements in patient identification, along with procedural and technical improvements especially for patients undergoing TAVR but also for SAVR given the contemporary mini-thoracotomy approach utilized in many patients. Subsequently, there has been a vast amount of data from both large, randomized trials and smaller cohort studies that have evaluated the outcomes of these patients. Some level of clinical equipoise has been noted between these two techniques and cardiovascular societies have been engaged in improving the adverse clinical outcomes of TAVR and SAVR including reduction in post-procedural stroke, cardiovascular and all-cause mortality. TAVR being a minimally invasive technique, appears to be an attractive alternative and is now considered the treatment of choice for patients with prohibitive surgical risk, and an equivalent option for high surgical risk. TAVR is also considered an alternative option for intermediate surgical risk patients and recently approved for selected low surgical risk patients from the U.S Food and Drug Administration (FDA) as well [8, 9]. The threshold of performing TAVR is gradually declining with ongoing advancement in operator experience, technique and valvular specifications. Cost effective analysis also supports TAVR in selected patient population as demonstrated by Weintraub et al. TAVR is comparable to medical therapy (PARTNER B sub study) and comparable to SAVR if trans-femoral approach is considered (PARTNER A sub study) [10].

Periprocedural stroke remains a dreaded complication for both procedures and, the literature demonstrates conflicting evidence for this adverse outcome. Unfortunately, over nearly two decades, we were not able to appreciate any temporal decline in postprocedural stroke at 1-year despite lower rates of preexisting AFib in more recent studies. Although the incidence of stroke/TIA at 1-year was higher in TAVR in first tertile of our study period, incidence of stroke/TIA at 1 year in TAVR was lower compared to SAVR population in the latter tertiles (Fig. 4). Given the fact that despite progressively less preexisting AFib in SAVR population, there is more incidence of post-procedural strokes indicating, in aggregate, increased risk with this procedure. In clinical practice, anticoagulation for peri-operative AFib post-SAVR is often neglected due to the misconception that AFib is incidental and purely catecholamine induced, although recurrent AFib is common and could be responsible for these events [11–14]. Chakravarty et al. described the effect of short term (up to 1 year) anticoagulation post-SAVR population with decreased risk of stroke post-procedure [15]. Given this data, it is important to note that peri-operative AFib needs to be considered as a potential driver of these findings.

On the contrary, structural valve degeneration (SVD) remains an area of concern after TAVR and the longevity of these prosthetic valves needs to be considered in the decision making especially in patients with a life expectancy of more than 5 years. A recent study from the UK assessed the durability of TAVR up to 10 years in 241 patients and found > 90% to remain free of SVD and < 1% to suffer from severe SVD [16]. Another study evaluating high surgical risk patients undergoing TAVR from the FRANCE-2 registry found the rate of severe SVD and moderate to severe SVD at 5 year to be 2.5% and 13.3% respectively [17]. Five-year outcomes from high-risk patients enrolled in PARTNER 1 trial showed no SVD requiring surgical valve replacement in both TAVR and SAVR population, however, moderate-to-severe aortic regurgitation rate was higher in TAVR compared to SAVR (14% vs. 1%) and, when present, associated with worse mortality [18]. Five-year outcome from PARTNER 2 demonstrated that more patients in TAVR group had at least mild paravalvular aortic regurgitation (33.3% vs. 6.3%), as were aortic-valve reinterventions at 5 years, there was no significant difference in the incidence of death from any cause or disabling stroke between the TAVR group and the surgery group (47.9% and 43.4%) [19]. On the contrary, in the low surgical risk NOTION trial, at 6 year follow up, moderate-to-severe SVD was higher in SAVR population compared to TAVR population (24% vs. 4.8%) [20]. Moreover, emerging data on newer generation valves is revealing very low incidence of SVD up to at least 5 years following the intervention [21]. This evidence does support the efficacy of TAVR for the intermediate term, but more studies are required to validate these findings and to determine the long-term durability of TAVR compared to SAVR.

Accordingly, our meta-analysis provides the conceptualized framework for temporal changes in hard outcomes over the previous 15–20 years due to the constant evolution of technological advances. It represents the paradigm shift of post procedural stroke at 1 year over the decade favoring TAVR over SAVR populations. It also provides the framework for evidence to support the current practice for giving preference to TAVR for prohibitive and high surgical risk patients, while validating its applicability in intermediate and selected low surgical risk populations due to comparable all-cause and cardiovascular mortality with SAVR population.

Importantly, rehospitalization rates were higher in the SAVR compared to TAVR population and one needs to consider the potential increase burden in healthcare utilization from this aspect. The economic evaluation of medical technologies is usually performed by cost-effectiveness analysis. Quality adjusted life years (QALYs) and incremental cost effectiveness ratio (ICER) are two valuable parameters used for this purpose as described by Baron et al. [22]. The American Heart Association and American College of Cardiology have created a general guideline in which an ICER < $50,000/QALY gained is considered to be of a high economic value, $50,000 to $150,000/QALY is considered to be of an intermediate value and an ICER > $150,000/QALY is considered to be of a low valve in US healthcare system [23]. As demonstrated by Baron et al., TAVR was cost-effective in prohibitive and high-risk populations, and cost-saving in intermediate risk population, despite high procedural cost of TAVR compared to SAVR [22]. Meduri et al. exhibited a similar analysis that when taking all costs into account; overall there are comparable expenditures in both TAVR and SAVR groups. Similarly, Baron et al. demonstrated that TAVR is estimated to be beneficial in providing grater quality adjusted life expectancy along with lower long-term cost for the US healthcare system [24].

## Limitations

Our study has certain limitations. We could not control the inherent variations among the included studies including baseline characteristics, different vascular access, type of the implanted valves and different expertise of centers where these procedures were performed. Similarly, temporal trend analyses always have limitations. However, in order to incorporate the inherent limitations utilizing a meta-analysis, a far more sophisticated computation approach using ‘R program meta’ was herein utilized with the guidance of two mathematicians/ statisticians (GR and MD). We note that there is always a chance of publication bias in meta-analysis. Here, Abramowitz et al. contributed a larger number of patients even though that was not clearly seen on our funnel plot analysis [25]. Another inherent limitation of our meta-analysis is that we were not able to incorporate data at the individual patient level, but in study groups based on the published studies, thus possibly underestimating the temporal changes between these procedures. This is a well understood limitation not unique to our analysis. Also, very few studies had reported comparison between TAVR and SAVR data hence certain statistics could not be applied for entire population. The cohorts were not stratified by risk and year combined as we did not have enough patient population to perform accurate analysis. We did not include other adverse clinical outcomes such as paravalvular leak, concomitant (other than SAVR) cardiothoracic intervention, permanent pacemaker implantation or vascular complications which may play a role in the surgical decision over either of these modalities. Also, data on new onset AFib post procedural were not reported in all the studies. Although our study provides considerable of insightful information for early to intermediate outcomes, we are unable to comment on the long-term outcomes for valve durability and degeneration.

## Conclusion

While there is a trend for overall improvement over nearly 2 decades in both TAVR and SAVR outcomes, our integration of over 65,000 patients incorporating 23 studies, in aggregate, failed to robustly demonstrate that either outperforms the other when it comes to hard short-term outcomes, supporting no distinct separation in all-cause mortality and adjudicated cardiovascular mortality at 1-year between groups except for the highest surgical risk population who shared lower post procedural stroke, cardiovascular and all-cause mortality at 1-year. We propose that this has substantial socio-economic implications as we contemplate further expansion of our TAVR indications to low/intermediate risk populations.

## Data Availability

The datasets used and/or analyzed during the current study are available from the corresponding author on reasonable request.

## Abbreviations

AS: Aortic stenosis
TAVR: Transcatheter aortic valve replacement
SAVR: Surgical aortic valve replacement
AFib: Atrial fibrillation
PRISMA: Preferred Reported Items for Systematic Reviews and Meta-analyses
RCT: Randomized clinical trial
ROBIN-I: Risk Of Bias In Non-Randomized Studies of Interventions
STS: Society of Thoracic Surgeons
EuroSCORE: European System for Cardiac Operative Risk Evaluation
RR: Relative risk
FDA: Food and Drug Administration
SVD: Structural valve degeneration
QALYs: Quality adjusted life years
ICER: Incremental cost effectiveness ratio
